# Stressful parental bonding exaggerate the functional and emotional disturbances of primary dysmenorrhea

**DOI:** 10.3402/ejpt.v5.26532

**Published:** 2014-12-09

**Authors:** Kai Xu, Liuxi Chen, Lingyun Fu, Hongjing Mao, Jian Liu, Wei Wang

**Affiliations:** Department of Clinical Psychology and Psychiatry, School of Public Health, Zhejiang University College of Medicine, Hangzhou, People's Republic of China

**Keywords:** parental bonding, primary dysmenorrhea, trauma, attachment, family relationships, painful period, emotional disturbance

## Abstract

**Background:**

Some evidence suggests that women with primary dysmenorrhea (or painful period) often have traumatic experience with parental attachments, but the exact relationship between styles of the parental bonding and the detailed aspects of the disorder is unclear.

**Methods:**

From university-student women, we invited 50 primary dysmenorrhea patients and 111 healthy volunteers to undergo tests of the functional and emotional measure of dysmenorrhea (FEMD), the Family Relationship Questionnaire (FRQ), and the visual analog scale for the pain intensity experienced.

**Results:**

Besides the high scores of the FEMD functional and emotional scales, the dysmenorrhea patients also scored significantly higher than the healthy controls on the FRQ scales of paternal dominance and maternal abuse. In patients, the FEMD Emotional scale was negatively predicted by the Paternal Freedom Release scale and the FEMD functional scale was positively predicted by the Maternal Dominance scale.

**Conclusion:**

Inappropriate parental bonding or chronic traumatic attachment styles have respective relationships with the functional and emotional disturbances experienced by the primary dysmenorrhea patients.

